# Torpor‐assisted migration: Regular use of shallow torpor reduces rest‐phase energy expenditure for songbirds during migration

**DOI:** 10.1111/1365-2656.70244

**Published:** 2026-03-30

**Authors:** Ryan A. C. Leys, Lily Hou, Liam P. McGuire

**Affiliations:** ^1^ Department of Biology University of Waterloo Waterloo Ontario Canada

**Keywords:** energetics, heterothermy, metabolic rate, migration, physiology, stopover, torpor

## Abstract

1. Endothermy evolved independently in birds and mammals providing many fitness benefits but requiring substantial energy to maintain a stable body temperature. Many endotherms have evolved heterothermic strategies to cope with daily and seasonally cold weather and reduced food availability.
2. Heterothermy has been studied extensively in mammals, but comparatively few studies have considered heterothermy in birds. Heterothermy may be beneficial to migrating endotherms to reduce thermoregulatory energy expenditure and enhance refuelling rate at stopover sites, known as torpor‐assisted migration.
3. We hypothesized that migratory birds use shallow torpor to reduce rest‐phase energy expenditure. We tested four species for shallow torpor use during migration: Brown Creeper *Certhia americana*, Golden‐crowned Kinglet *Regulus satrapa*, Ruby‐crowned Kinglet *Corthylio calendula* and Yellow‐rumped Warbler (Myrtle subspecies) *Setophaga coronata coronata*. We measured body temperature using temperature‐sensitive PIT tags and metabolic rate using open‐flow respirometry at night.
4. All four species from three different passerine families (Regulidae, Certhiidae and Parulidae) used shallow torpor frequently (69%–98% of birds per species) over a range of ambient temperatures (−1 to 30°C) during migration. Body condition was the main driver of torpor use. Birds with smaller fat stores used torpor more deeply and frequently than fatter birds, achieving up to 42% lower metabolic rates and saving more energy. Contrary to studies on deep torpor, ambient temperature had no effect on the frequency or depth of torpor use.
5. Shallow torpor has wide‐ranging implications for the energetics of migration with broader impacts on survival and reproduction and provides an opportunity to compare heterothermic strategies between birds and mammals.

## Graphical abstract

Migrating songbirds regularly use shallow torpor (up to 98% of birds for some species) to save energy (up to 42%) during inactive periods. This Ruby‐crowned Kinglet *Corthylio calendula* that was captured after a spring snowstorm highlights the value of being able to save energy when encountering unfavourable conditions. We show that four species of songbirds can reduce their body temperature and metabolic rate at night (top graph), and that the frequency and depth torpor use is driven by body condition. Metabolic rate increases at lower ambient temperatures but is reduced at lower body temperatures (bottom graph). The top graph represents data from a Yellow‐rumped Warbler (Myrtle subspecies) *Setophaga coronata coronata* at 2°C, and the bottom graph represents data from Myrtle Warblers in respirometry. Yellow‐rumped Warbler photo by Brock & Sherri Fenton.

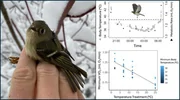

## 1 INTRODUCTION

Endothermy has independently evolved twice among vertebrates, in birds and mammals (Clarke & Pörtner, 2010; Hulbert & Else, 2000). Endothermy provides many fitness benefits (Clarke & Pörtner, 2010; Hulbert & Else, 2000) but is energetically costly, especially for small‐bodied endotherms that rapidly lose heat to the environment because of a relatively higher surface area to volume ratio. To maintain a stable body temperature (*T*_b_), small endotherms must increase metabolic heat production to offset increased heat loss (Wooden & Walsberg, 2002).

Many birds and mammals adaptively use heterothermic thermoregulatory strategies (controlled reductions of *T*_b_ and metabolic rate) to cope with high energetic costs of endothermy. Endothermic costs are particularly pronounced in temperate regions due to seasonally cold ambient temperatures (*T*_a_) and seasonally low resource availability (Ruf & Geiser, 2015). Many endotherms that are unable to migrate (e.g. most small mammals; Webber & McGuire, 2022) use daily torpor or hibernate for several months to minimize energy costs and increase fitness in unfavourable environments (Geiser, 2004). During deep torpor, resting metabolic rate may be reduced to <5% compared to homeothermic values and *T*_b_ reduced to within a few degrees of *T*_a_, yielding energy savings >90% compared to homeothermic resting metabolic rate (Geiser, 2004; McGuire et al., 2014). It is now well understood that daily torpor is not simply a response to energetic stress and there are many adaptive functions of torpor (Geiser & Brigham, 2012; Nowack et al., 2017). For example, torpor can be used to slow foetal development and avoid giving birth in unfavourable environmental conditions (Willis et al., 2006), and torpor can be an important aspect of water conservation in arid zone species (Noakes et al., 2022; Ruf & Geiser, 2015).

Compared with the extensive and diverse literature describing heterothermic responses in mammals, heterothermic strategies in birds have received comparatively little attention (but see Clerc & McGuire, 2021). Hummingbirds use deep torpor to survive at high altitudes (Wolf et al., 2020), and many caprimulgids use deep torpor for various purposes (Nowack et al., 2017), but heterothermy is thought to be uncommon in birds (McKechnie et al., 2023; McKechnie & Lovegrove, 2002). This is especially the case for passerines, representing approximately 60% of extant bird species (Raikow, 1986; Schmitt & Edwards, 2022). Deep torpor has not been reported in this group, but there is increasing recognition that passerines can use shallow torpor to conserve energy (Benedetti et al., 2014; Maddocks & Geiser, 1997; McKechnie & Lovegrove, 2002; Wojciechowski & Pinshow, 2009). Shallow torpor is characterized by a controlled, intermediate lowering of *T*_b_ and metabolic rate (McGuire et al., 2023), which can lead to substantial energy savings compared to normothermic rates (Clerc & McGuire, 2021). For example, Silvereyes *Zosterops lateralis* that reduced *T*_b_ by 5.5°C at night achieved a concurrent reduction in metabolic rate of up to 50% (Maddocks & Geiser, 1997).

Approximately 20% of bird species are migratory (Kirby et al., 2008), increasing to >80% in temperate regions (Torstenson et al., 2024). Migratory flight is extremely energetically demanding (Guglielmo, 2010), but theoretical models and empirical evidence suggest birds expend twice as much energy during periods of stopover, primarily driven by thermoregulatory costs (Hedenström & Alerstam, 1997; Wikelski et al., 2003). At lower *T*_a_, an increasing fraction of ingested energy is used for thermoregulation rather than stored as fat to fuel migratory flight (Cooper, 2000; Guglielmo, 2010, 2018). To mitigate this challenge, migrating bats and hummingbirds regularly enter deep torpor during migration, reducing resting energy expenditure by up to 91% compared to remaining homeothermic (Carpenter & Hixon, 1988; Eberts et al., 2021; McGuire et al., 2014). This strategy, known as torpor‐assisted migration (McGuire et al., 2014), allows migratory endotherms to reduce rest‐phase energy expenditure, increase refuelling rate, make shorter stopovers and increase the overall speed of migration (McGuire et al., 2023), which incurs fitness benefits during the deadliest period of the annual cycle (Sillett & Holmes, 2002). Therefore, shallow torpor could provide a thermoregulatory strategy for migratory passerines to overcome energetic challenges experienced at stopover; however, to our knowledge, no studies have assessed the prevalence of shallow torpor across multiple species, and few studies have examined heterothermic strategies in migratory birds or the intrinsic and extrinsic factors that drive shallow torpor use (but see Benedetti et al., 2014; Wojciechowski & Pinshow, 2009).

Our research objectives were to assess the prevalence of heterothermy in migrating passerines, determine how intrinsic and extrinsic factors influence torpor use, and to describe the energetic implications at stopover sites. We hypothesized that migrating birds can mitigate the high energetic cost of thermoregulation during the rest phase of stopover by using shallow torpor. Specifically, we predicted that: (1) passerines would use shallow torpor to reduce the high energetic cost of low *T*_a_, (2) frequency and depth of torpor use would be inversely related to *T*_a_ and body condition, (3) birds that use shallow torpor would reduce energy expenditure and (4) shallow torpor would result in reduced overnight rate of mass loss.

## 2 MATERIALS AND METHODS

### 2.1 Study species and field methods

Our focal species were Brown Creeper *Certhia americana*, Golden‐crowned Kinglet *Regulus satrapa*, Myrtle Warbler (subspecies of Yellow‐rumped Warbler) *Setophaga coronata coronata* and Ruby‐crowned Kinglet *Corthylio calendula*. We selected these four species as we predicted they were likely to use shallow torpor based on small body size (large surface area to volume ratio), early spring/late fall migration timing (colder *T*_a_) and an insectivorous diet (reduced prey availability and abundance in cold weather; Dunn et al., 2023; Greenberg & Forrest, 2003; Kontopoulos et al., 2025; McGuire et al., 2023; Vercher et al., 2023; Williams, 1961).

We captured birds at the Long Point Bird Observatory (Long Point, Ontario, Canada; 42°35′ N, 80°24′ W) during spring and fall migration. Our study site is along the migration route for each species, with the core breeding range to the north and the core wintering range to the south of our study site (Hunt & Flaspohler, 2020; Poulin et al., 2020; Swanson et al., 2020, 2021). All birds were captured in the 3 h prior to sunset. We recorded age, sex, wing chord (±1 mm), body mass (m_b_; ±0.1 g; ACCULAB VIC‐711) and fat score (8‐point scale). One author (R.A.C.L.) collected all biometric data. We did not provide food or water because the birds were held during their natural rest period and released shortly after sunrise to continue their natural foraging. We assigned birds to one of two age groups: juvenile (had not yet completed a full migration cycle) or adult (had completed at least one full migration cycle) because age has been shown to affect minimum nocturnal *T*_b_ and metabolic rate (Andreasson et al., 2019; McCabe & Guglielmo, 2019). We defined a full migration cycle as migrating to the wintering grounds and returning to the breeding grounds the following year. We tested our predictions by conducting two studies: Study 1—birds held overnight in an outdoor aviary and Study 2—birds held overnight in respirometry chambers for measurement of metabolic rate.

#### 2.1.1 Study 1: Overnight body temperature in an outdoor aviary

We captured birds between 15 April–17 May 2021, and 2 April–8 May 2022. We balanced age and sex ratios when possible with the exception of Brown Creeper, which cannot be reliably aged or sexed in hand, except in limited circumstances (Pyle, 1997). We injected temperature‐sensitive passive integrated transponder (PIT) tags (Biotherm13, 25–45°C ± 0.5°C, Biomark, Boise, Idaho) subcutaneously between the scapulae following established methods (Hou et al., 2015; Nicolaus et al., 2008). After a 5 min waiting period, we placed birds in individual cages (17.8 cm H × 12.7 cm diameter) located in an outdoor, predator‐proof aviary with an antenna around the bottom of each cage connected to a datalogging reader (HPR Plus; Biomark, Boise, Idaho, USA). The readers recorded PIT‐tag temperature (*T*_pit_) every ~10 s starting <2.5 h before sunset until <1 h after sunrise. We recorded *T*_a_ at 10‐min intervals with a calibrated iButton (±0.5°C; Embedded Data Systems, Lawrenceburg, KY, USA) placed in the aviary. We recorded m_b_ and fat score prior to release in the morning. Subcutaneous *T*_pit_ is an accurate proxy of *T*_b_ in small birds (<20 g) across *T*_a_ from −15 to 40°C (Andreasson et al., 2023; Oswald et al., 2018). We tested this assumption in our study species (<15 g) by simultaneously recording *T*_pit_ and cloacal temperature (*T*_cloacal_) on a subset of birds (*n* = 12: 9 Myrtle Warbler, 2 Golden‐crowned Kinglet, 1 Ruby‐crowned Kinglet) prior to release in the morning, but we did not record *T*_a_ during comparative *T*_b_ measurements. We recorded *T*_cloacal_ with a digital thermometer (Extech Instruments, Nashua, New Hampshire, USA) with a Type‐K thermocouple (±0.1°C).

#### 2.1.2 Study 2: Overnight body temperature and metabolic rate in a metabolic chamber

We captured birds between 21 September–2 November 2021 and 29 September–27 October 2022. We placed PIT‐tagged birds in 1 L respirometry chambers in a temperature‐controlled cabinet (modified 204 L chest freezer; Danby Premier) and controlled temperature (±0.5°C) using a temperature controller (Model ITC‐308, Inkbird) with the freezer plugged into the cooling socket and a small space heater (Heatbud Mini‐Ceramic Heater, Honeywell) plugged into the heating socket. We placed a PIT‐tag antenna around the bottom of each chamber to simultaneously record *T*_b_ and metabolic rate. We exposed birds to one of four temperature treatments: 2°C, 7°C, 12°C and either 25°C (Brown Creeper and Myrtle Warbler) or 30°C (Golden‐crowned Kinglet and Ruby‐crowned Kinglet). We also tested two Ruby‐crowned Kinglets at 25°C. We selected 2, 7 and 12°C as representative of *T*_a_ during fall migration at our field site, and 25 and 30°C as representative of the lower bounds of the allometrically predicted thermoneutral zone for each species (Aschoff, 1981).

We used open‐flow respirometry to measure reductions in metabolic rate associated with shallow torpor following established methods (McGuire et al., 2014). We scrubbed incurrent air of H_2_O using Drierite, CO_2_ using soda lime, and H_2_O again using magnesium perchlorate. We maintained airflow at ~300 mL min^−1^ using a mass flow meter (Flowbar FB‐8, Sable Systems International, Las Vegas, Nevada, USA). We scrubbed excurrent air of H_2_O using magnesium perchlorate prior to CO_2_ and O_2_ measurements. We measured incurrent and excurrent CO_2_ and O_2_ concentration using a Field Metabolic System (Sable Systems International, Las Vegas, Nevada, USA) and collected and analysed data using ExpeData software. We lag and drift corrected fractional concentrations of CO_2_ and O_2_ and used equations 11.7 and 11.8 from Lighton (2019) to calculate mean $\dot{V}{\mathrm{CO}}_{2}$ (mL min^−1^) and $\dot{V}O_{2}$ (mL min^−1^) for the lowest 30 s in the last 120 s of each 10‐min cycle to allow for equilibration and system washout. We cycled between birds and a baseline channel such that metabolic rate of each bird was sampled every 20–80 min. We recorded m_b_ and fat score prior to release.

### 2.2 Statistical analysis

#### 2.2.1 Shallow torpor use

We compared simultaneous measurements of subcutaneous *T*_pit_ and *T*_cloacal_ using a paired *t*‐test to determine if subcutaneous *T*_pit_ is an accurate proxy of *T*_b_ in small birds. To identify periods of shallow torpor, we calculated mean species‐level active phase *T*_b_ by examining *T*_b_ graphs for each bird and selecting birds with a stable *T*_b_ for >15 min in the morning (*n* = 15 Brown Creeper, 15 Golden‐crowned Kinglet, 14 Myrtle Warbler, 36 Ruby‐crowned Kinglet) and calculated mean *T*_b_ of the stable period. Next, we subtracted the allometrically predicted daily normothermic *T*_b_ oscillation range (Aschoff, 1981) from mean active phase *T*_b_ to determine the shallow torpor threshold for each species.

We pooled data from Study 1 and Study 2 and then used logistic regression to investigate which variables influenced shallow torpor use. The initial model included age, sex, fat score, mean *T*_a_, species and study (to account for methodological or seasonal effects). Brown Creeper models did not include age or sex for the reason mentioned above. We used mean night *T*_a_ for birds in Study 1 and temperature treatment as *T*_a_ for birds in Study 2. We followed a backwards stepwise model selection procedure until only significant terms remained (Zuur et al., 2009).

#### 2.2.2 Minimum body temperature and metabolic rate

We used separate linear models for each species to investigate which variables influenced minimum *T*_b_ and metabolic rate. Initial models included age, sex, fat score, *T*_a_, study and all two‐way interactions. The initial metabolic rate models also included minimum *T*_b_ and day of year. Model selection proceeded as described above.

#### 2.2.3 Rate of mass loss

We determined the rate of mass loss (g h^−1^) from evening and morning m_b_. We used linear regression models to investigate the rate of mass loss for each species separately. Models initially included age, sex, mean *T*_a_, proportion of time using torpor, study and all two‐way interactions. Model selection proceeded as described above.

We used the Benjamini–Hochberg procedure to compute a global threshold (*α* = 0.034) to control for the false discovery rate (Benjamini & Hochberg, 1995). We conducted all statistical analyses in RStudio version 2026.01.1+392 (Posit Team, 2026) with the statistical software R version 4.3.3 (R Core Team, 2025). All procedures were approved by the University of Waterloo Animal Care Committee (Protocol #43009) and Environment and Climate Change Canada (Permit #10169 CM).

## 3 RESULTS

We captured 44 Brown Creepers, 66 Golden‐crowned Kinglets, 59 Myrtle Warblers and 95 Ruby‐crowned Kinglets (*n* = 264) and obtained useable PIT‐tag data from 43 Brown Creepers, 58 Golden‐crowned Kinglets, 52 Myrtle Warblers and 86 Ruby‐crowned Kinglets (*n* = 239; Table S1). The species‐specific values for mean m_b_, allometrically predicted normothermic *T*_b_ oscillation range, *T*_b_ range and mean active phase *T*_b_ are reported in Table 1. We found no difference between *T*_cloacal_ and *T*_pit_ (*t*_21.7_ = −0.04, *p* = 0.97) indicating that subcutaneous *T*_pit_ is a reliable measure of *T*_b_ in small (<15 g) birds. The mean nocturnal *T*_a_ during spring migration ranged from −1 to 18.5°C for birds in the outdoor aviary.

**TABLE 1:** Allometrically predicted *T*_b_ oscillation range, body mass, observed *T*_b_ range (minimum and maximum observed *T*_b_ across all birds from both studies) and mean active phase *T*_b_ in our study species. Mean values reported ±SE for mass and active phase *T*_b_.

	Brown Creeper	Golden‐crowned Kinglet	Myrtle Warbler	Ruby‐crowned Kinglet
Allometrically predicted *T*_b_ oscillation range (°C)	3.2	3.3	3.0	3.3
Body mass (g)	8.6 ± 0.1	6.5 ± 0.1	12.7 ± 0.2	7.1 ± 0.1
Observed *T*_b_ range (°C)	34.1–43.8	28.8–43.0	33.4–43.5	34.5–43.6
Active phase *T*_b_ (°C)	41.5 ± 0.1	40.8 ± 0.2	42.0 ± 0.1	41.4 ± 0.1

### 3.1 Shallow torpor use

All four of our study species regularly used shallow torpor during the rest phase (Figure 1). Birds in better body condition were less likely to use shallow torpor (*β* = −0.78, SE = 0.17, *z* = −4.51, *p* < 0.0001). Specifically, each point increase on the fat score scale was associated with a 46% reduction in the likelihood of torpor use (95% CI: 32%–63%, *p* < 0.0001). The frequency of torpor use was not affected by age, sex, *T*_a_ or study (*all p* > 0.05).

**FIGURE 1:**
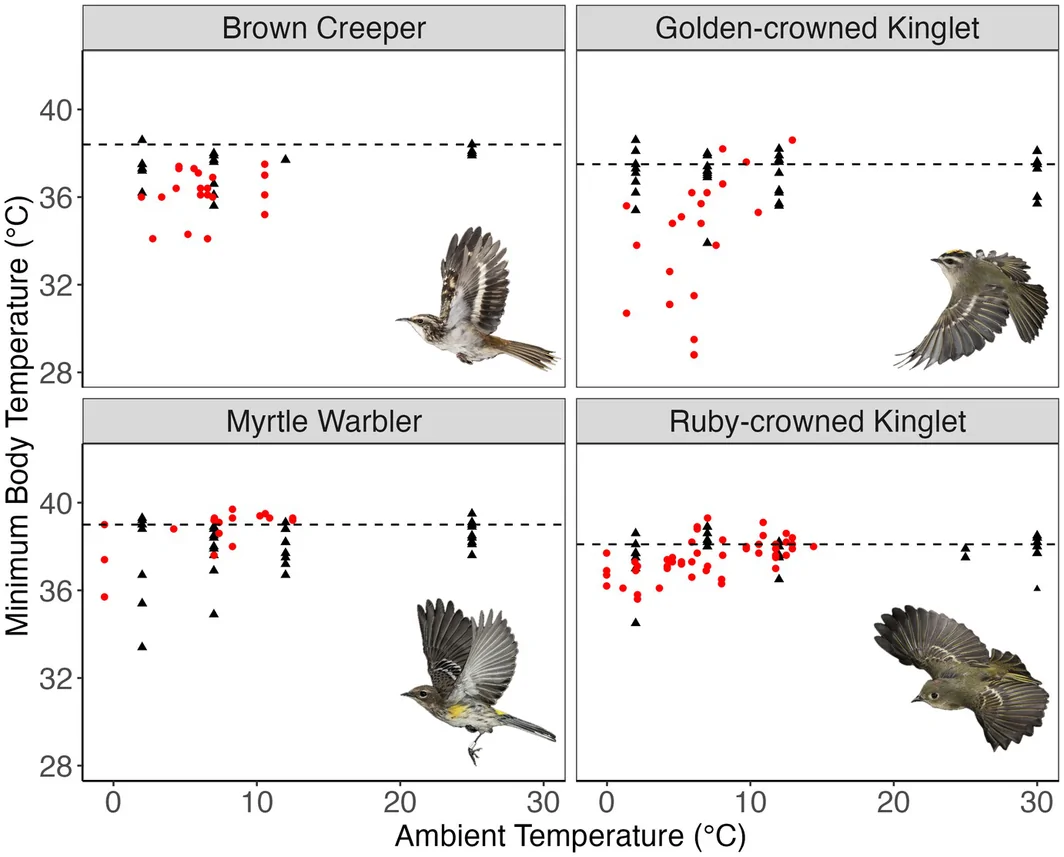
We observed shallow torpor in 98% (42/43) of Brown Creepers, 72% (42/58) of Golden‐crowned Kinglets, 69% (36/52) of Myrtle Warblers and 73% (63/86) of Ruby‐crowned Kinglets. The lowest recorded *T*_b_ was 28.8°C by a Golden‐crowned Kinglet. The dashed line in each figure indicates the threshold used to delineate shallow torpor from euthermia (allometric prediction of the lower bound of daily body temperature oscillation). Red circles represent birds in Study 1 (outdoor aviary), black triangles represent birds in Study 2 (respirometry). Each point represents a unique bird. Photos by Brock and Sherri Fenton.

### 3.2 Body temperature

Lower fat score was associated with lower minimum *T*_b_ in Brown Creeper (*F*_1,39_ = 11.7, *p* = 0.001), Myrtle Warbler (*F*_1,50_ = 12.7, *p* < 0.001) and Ruby‐crowned Kinglet (*F*_1,82_ = 11.6, *p* = 0.001). For Golden‐crowned Kinglet, the effect of fat score on minimum *T*_b_ differed between Study 1 and 2 (*F*_1,54_ = 14.6, *p* < 0.001); therefore, we parsed the data between the two studies and re‐ran the models. Fat score had no effect on Golden‐crowned Kinglet minimum *T*_b_ in respirometry (*F*_1,35_ = 2.1, *p* = 0.16), while birds with lower fat scores had lower *T*_b_ in the aviary (*F*_1,29_ = 14.6, *p* = 0.001). Minimum *T*_b_ was not affected by age, sex or *T*_a_ in any species (all *p* > 0.06; full model results in Table S2).

### 3.3 Metabolic rate

For Study 2, we captured 24 Brown Creepers, 41 Golden‐crowned Kinglets, 40 Myrtle Warblers and 42 Ruby‐crowned Kinglets (*n* = 147) and collected sufficient PIT‐tag and metabolic rate data from 23 Brown Creepers, 37 Golden‐crowned Kinglets, 35 Myrtle Warblers and 37 Ruby‐crowned Kinglets (Figure 2; *n* = 132; Table S1). Metabolic rate means and ranges are reported in Table S3. Fat score and body mass were positively correlated for all species: Brown Creeper (Pearson's *r* = 0.66, *p* < 0.001), Golden‐crowned Kinglet (Pearson's *r* = 0.59, *p* < 0.001), Myrtle Warbler (Pearson's *r* = 0.58, *p* < 0.001) and Ruby‐crowned Kinglet (Pearson's *r* = 0.44, *p* = 0.006); therefore, we included fat score and not body mass in all models because we were specifically interested in fat as the main fuel source.

**FIGURE 2:**
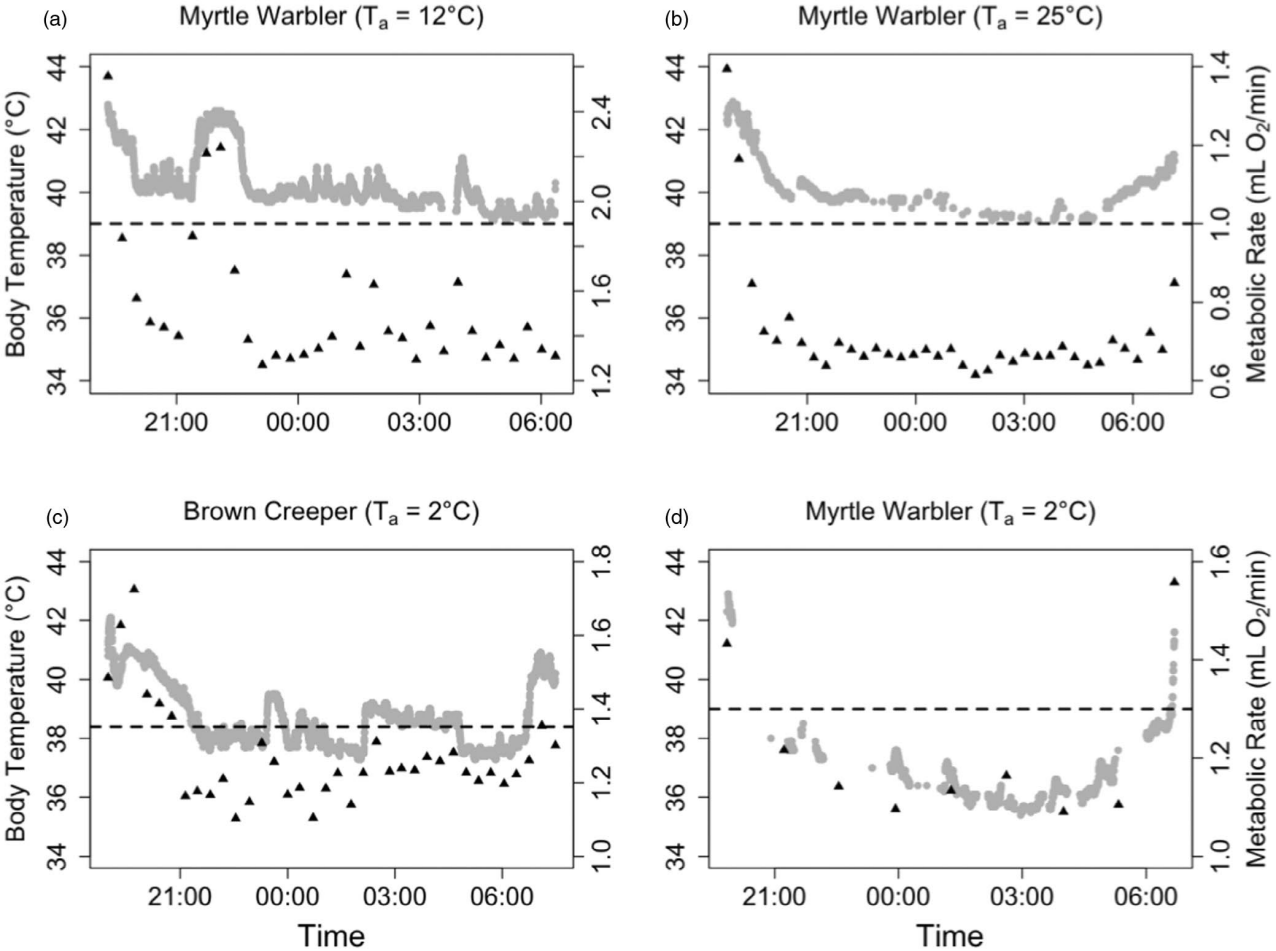
Representative simultaneous *T*_b_ and metabolic rate traces for four birds in respirometry. Panels (a, b) represent birds that were normothermic throughout the night. Figures (c, d) show birds that used shallow torpor. The Brown Creeper in panel (c) may have used multiple shorter bouts of torpor, with body temperature suddenly increasing above the torpor threshold at ~23:00 and ~02:00, whereas the Myrtle Warbler in panel (d) remained in a state of shallow torpor the entire night before rewarming to active *T*_b_ prior to sunrise. Grey circles represent *T*_b_, and black triangles represent metabolic rate. Horizonal dashed lines show the torpor threshold for each species (Brown Creeper = 38.4°C, Myrtle Warbler = 39.0°C).

Birds exposed to colder *T*_a_ had higher metabolic rate across all species (Figure 3; Brown Creeper: *F*_1,20_ = 299.2, *p* < 0.0001; Golden‐crowned Kinglet: *F*_1,33_ = 350.1, *p* < 0.0001; Myrtle Warbler: *F*_1,30_ = 573.8, *p* < 0.0001; Ruby‐crowned Kinglet: *F*_1,34_ = 260.7, *p* < 0.0001; full model results in Table S4). However, there was substantial variation in *T*_b_ at a given *T*_a_, with lower *T*_b_ associated with lower metabolic rate for Golden‐crowned Kinglet (*F*_1,33_ = 20.1, *p* < 0.0001), Myrtle Warbler (*F*_1,30_ = 42.3, *p* < 0.0001; Figure 4) and Ruby‐crowned Kinglet (*F*_1,34_ = 5.3, *p* = 0.028), but not Brown Creeper (*F*_1,19_ = 0.2, *p* = 0.69). Birds with higher fat scores had higher metabolic rates in Brown Creeper (*F*_1,20_ = 13.4, *p* = 0.002) and Myrtle Warbler (*F*_1,30_ = 28.4, *p* < 0.0001), but not in Golden‐crowned Kinglet (*F*_1,33_ = 4.2, *p* = 0.05) or Ruby‐crowned Kinglets (*F*_1,33_ = 1.3, *p* = 0.27). Phrased another way, birds in poorer condition (lower fat scores) reduced *T*_b_ to a greater extent, therefore having lower metabolic costs. Minimum metabolic rate decreased with the progression of fall migration in Myrtle Warbler (*F*_1,30_ = 12.4, *p* = 0.001). Minimum metabolic rate was not affected by age or sex in any species (all *p* > 0.50). The maximal metabolic rate reductions for all species ranged from 20% to 42%. Even at high *T*_a_, representing the lower end of the thermoneutral zone (25 and 30°C treatments), birds reduced metabolic rate by up to 31.1% and 40.9%, respectively.

**FIGURE 3:**
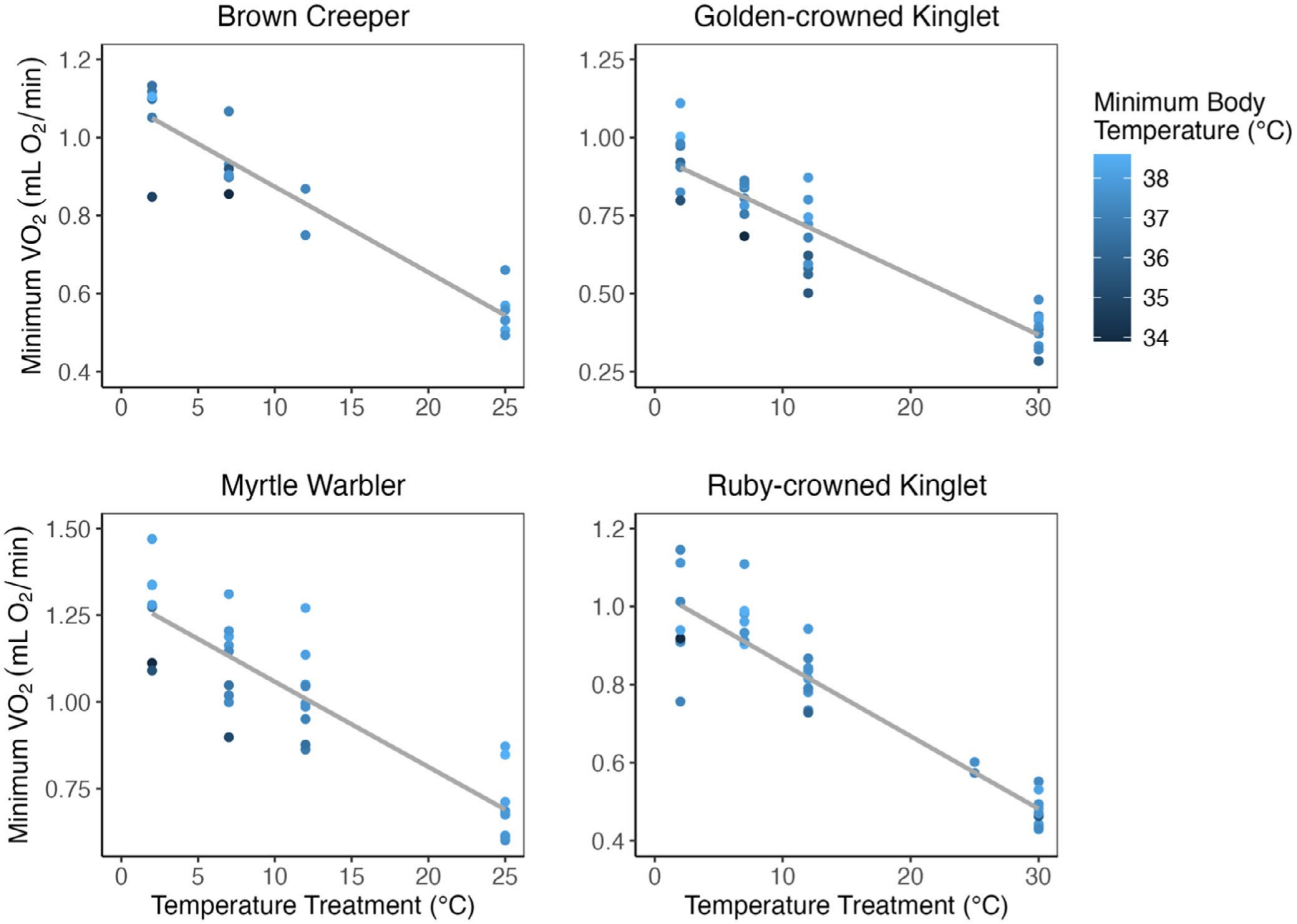
Minimum metabolic rate as a function of temperature treatment for each species. Minimum metabolic rate increased with decreasing *T*_a_ but decreased with lower *T*_b_ within each temperature treatment (see Figure 4). The temperature treatments were 2, 7, 12 and 25 or 30°C. Each point represents a unique bird and the colour scale represents the minimum *T*_b_ of each bird.

**FIGURE 4:**
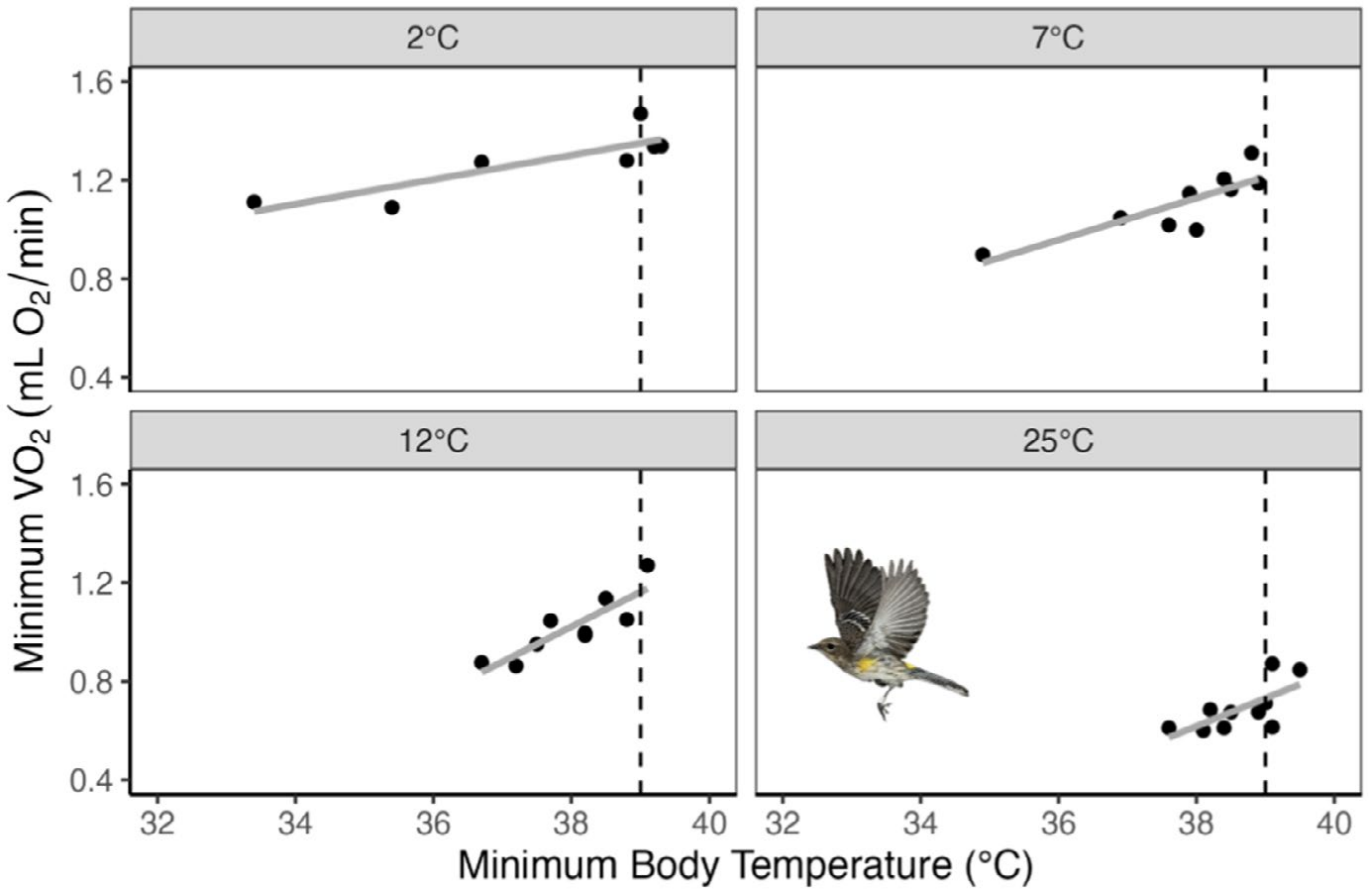
Birds with lower minimum *T*_b_ had lower minimum metabolic rate in each temperature treatment (data shown here from Myrtle Warblers). Each point represents a unique bird. The vertical dashed line represents the heterothermic threshold for Myrtle Warblers (39.0°C). The same relationship was observed for Golden‐ and Ruby‐crowned Kinglets.

### 3.4 Rate of mass loss

Mean overnight *T*_b_ and percentage of time using shallow torpor were negatively correlated for all species: Brown Creeper (Pearson's *r* = −0.74, *p* < 0.0001), Golden‐crowned Kinglet (Pearson's *r* = −0.84, *p* < 0.0001), Myrtle Warbler (Pearson's *r* = −0.85, *p* < 0.0001) and Ruby‐crowned Kinglet (Pearson's *r* = −0.76, *p* < 0.0001); therefore, we included percentage of time using shallow torpor in all models because percentage of time using shallow torpor has been shown to have a greater effect on energy savings than torpor depth (Shankar et al., 2020).

All species lost mass overnight (Brown Creeper: *t*_83.9_ = 5.0, *p* < 0.0001; Golden‐crowned Kinglet: *t*_113.5_ = 6.6, *p* < 0.0001; Myrtle Warbler: *t*_101.0_ = 4.0, *p* = 0.0001; Ruby‐crowned Kinglet: *t*_169.2_ = 8.8, *p* < 0.0001), accompanied by decreased fat score (Brown Creeper: *t*_77.5_ = 5.1, *p* < 0.0001; Golden‐crowned Kinglet: *t*_107.1_ = 4.9, *p* < 0.0001; Myrtle Warbler: *t*_100.3_ = 2.9, *p* = 0.004; Ruby‐crowned Kinglet: *t*_155.8_ = 7.5, *p* < 0.0001). Overnight mass loss ranged between 2.9% and 14.7% across all species.

Rate of mass loss was lower in Golden‐crowned Kinglets (*F*_1,19_ = 11.2, *p* = 0.003) and Ruby‐crowned Kinglets (*F*_1,83_ = 6.8, *p* = 0.01) that spent more time using shallow torpor, though the effect was only seen in Study 1 for Golden‐crowned Kinglets. For Myrtle Warblers, the relationship between sex and rate of mass loss varied between Study 1 and Study 2 (*F*_1,47_ = 8.8, *p* = 0.005); therefore, we ran separate models for each study. Females had a lower rate of mass loss than males in Study 2 (*F*_1,32_ = 6.2, *p* = 0.004), but sex did not affect the rate of mass loss in Myrtle Warbler in Study 1 (*F*_1,15_ = 1.2, *p* = 0.29). Age and *T*_a_ had no effect on the rate of mass loss in any species (all *p* > 0.10; full model results in Table S5). Minimum metabolic rate, independent of *T*_a_, did not affect the rate of mass loss in any species (all *p* > 0.046).

## 4 DISCUSSION

All four of our focal species used shallow torpor during migration with substantial reductions in *T*_b_ and metabolic rate overnight. Birds at lower *T*_a_ expended more energy on thermoregulation than conspecifics experiencing higher *T*_a_. From active *T*_b_ of 41–42°C, all birds maintained relatively high *T*_b_ (>28°C) compared to *T*_a_, which leads to increasing energy expenditure at decreasing *T*_a_ (Maggini & Bairlein, 2013), particularly for small‐bodied birds due to their high surface area to volume ratio (Wooden & Walsberg, 2002). Therefore, we expected to see more frequent and deeper torpor use at colder *T*_a_. However, *T*_a_ had no effect on frequency or depth of torpor use in any species, consistent with previous studies on shallow torpor in passerines (Maddocks & Geiser, 1997; Wojciechowski & Pinshow, 2009), but contrary to studies on deep torpor in non‐passerine birds (Brigham, 1992; Spence & Tingley, 2021; Wolf et al., 2020) and bats (McGuire et al., 2014) where *T*_b_ closely tracks *T*_a_. However, there was substantial intraspecific variation in metabolic rate among birds at the same *T*_a_. Birds with lower *T*_b_ had a 20%–42% lower metabolic rate (maximum reduction observed for each species) during the rest phase than normothermic birds indicating a substantial energy savings associated with using shallow torpor.

Body condition was the main driver of torpor use. Lean birds used shallow torpor more frequently and deeply than birds in better body condition, which suggests intrinsic factors (e.g. energetic status) have a greater influence on torpor use than extrinsic factors (e.g. *T*_a_). When using torpor, birds with lower fat stores had lower *T*_b_ and metabolic rates, consistent with previous studies on passerines during migration (Benedetti et al., 2014; Wojciechowski & Pinshow, 2009), but in contrast to migrating hummingbirds, which are more likely to use deep torpor with higher fat stores (Eberts et al., 2021). The fat score‐metabolic rate relationship was not observed in Golden‐ or Ruby‐crowned Kinglets. However, >86% (32/37) of Golden‐crowned Kinglets and >89% (33/37) of Ruby‐crowned Kinglets in respirometry were in good body condition (fat score ≥3), which may have masked the effect of body condition on metabolic rate. A facultative heterothermic migration strategy allows torpor‐assisted migrants to use the most favourable thermoregulatory strategy based on their current energetic condition. Birds in poor body condition can use shallow torpor to save energy by reducing the *T*_b_ set point to minimize the energetic cost of shivering thermogenesis required to maintain a high *T*_b_ (Hohtola, 2004), while birds with sufficient energy stores can maintain homeothermy to avoid the costs of torpor including the high energetic cost of rewarming (Geiser & Drury, 2003), oxidative stress (Buzadžić et al., 1997), sleep deprivation (Palchykova et al., 2002), reduced responsiveness to external stimuli (Wojciechowski & Pinshow, 2009), inhibited flight capability (Carr & Lima, 2013) and increased predation risk (Estók et al., 2009).

Insectivorous species face the additional challenge of reduced prey availability in cold weather (Dunn et al., 2023; Greenberg & Forrest, 2003; Williams, 1961). Decreased prey availability leads to a decrease in body condition for insectivorous and omnivorous migratory birds on their wintering grounds (Brown & Sherry, 2006; Strong & Sherry, 2000), even when not experiencing the additional energetic cost associated with migratory flight (Tatner & Bryant, 1986). Insectivorous species simultaneously facing cold *T*_a_ and reduced prey availability have a decreased energetic input and an increased energetic output, which may be further compounded by a compromised ability to rapidly increase fat mass upon arrival at stopover. Therefore, shallow torpor may be an especially important strategy for insectivorous species to manage their energy budget when facing environmental challenges.

While we did not study refuelling rate directly, Golden‐ and Ruby‐crowned Kinglets that used shallow torpor for a greater proportion of the rest phase had lower rates of mass loss. Shallow torpor may allow migrants to partially mitigate the challenges of variation in stopover site quality or environmental conditions and maintain a relatively higher net refuelling rate despite the conditions experienced at stopover (Figure 5; Clerc & McGuire, 2021). Heterothermic species may be better able to contend with harsher environmental conditions, allowing them to migrate northward earlier and southward later than homeothermic species. Further, the increased net refuelling rate associated with torpor‐assisted migration (Clerc & McGuire, 2021) may also reduce stopover duration (McGuire et al., 2023) compared to homeothermic species (Figure 5). Migrating earlier or at a faster pace allows migrants to reach breeding and wintering grounds more rapidly and potentially more safely (Sillett & Holmes, 2002), resulting in the fitness benefits of a longer breeding season (Møller, 1994), higher quality mate selection (Møller, 1994), increased territory quality (Aebischer et al., 1996) and reduced interspecific competition for food during migration (Moore & Yong, 1991). The benefits of a heterothermic migration strategy may be even further emphasized in birds that arrive at stopover sites with reduced digestive tract size and function (Hume & Biebach, 1996), even in short‐distance migrants that use a short‐hop migration strategy such as Myrtle Warblers (Lee et al., 2002). In the face of limited energy assimilation, torpor use may enhance survival in the nights immediately following a migratory flight and may facilitate a more rapid gain in fat stores, especially at low quality stopover sites where foraging opportunities may be limited.

**FIGURE 5:**
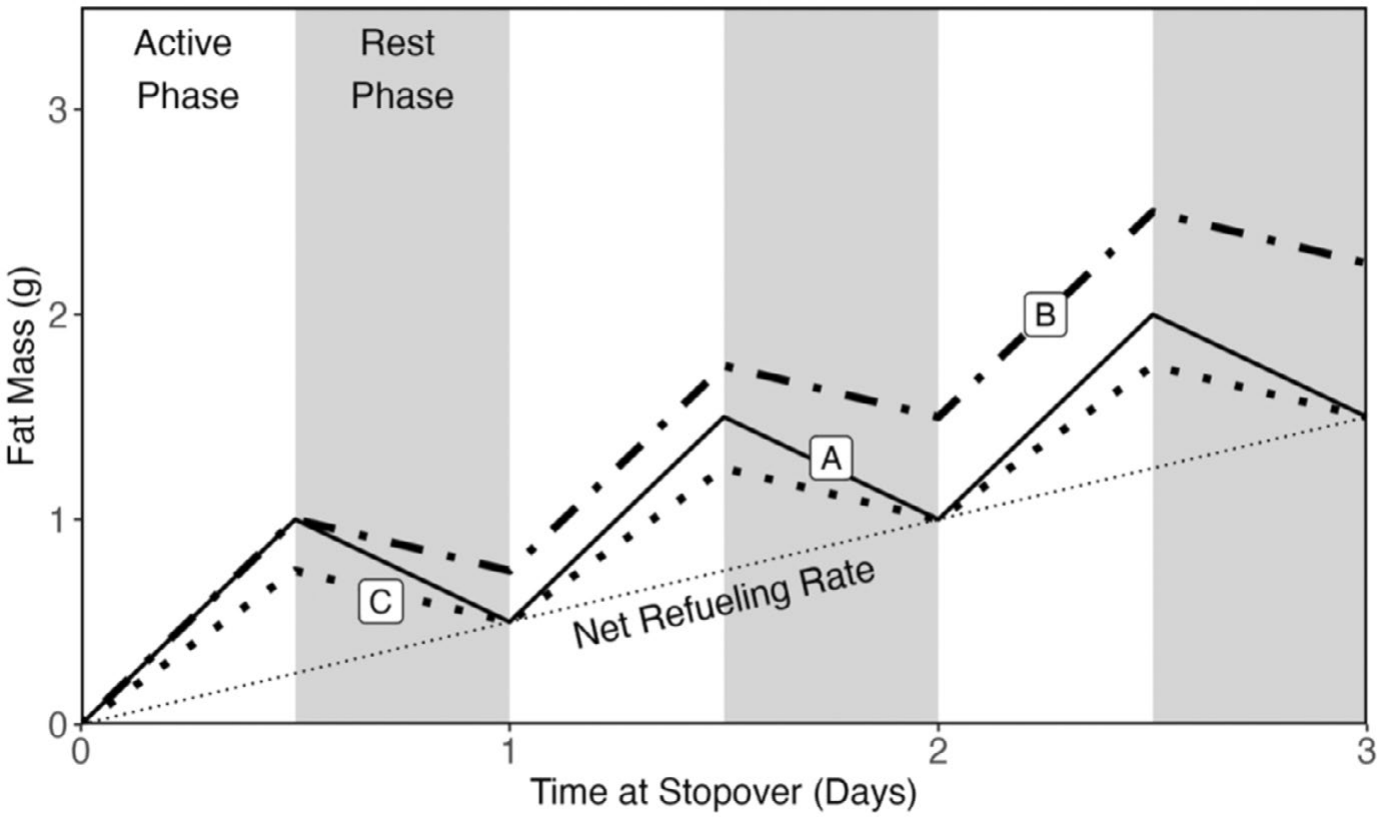
A conceptual model that illustrates the potential energy savings of shallow torpor during the rest phase. Homeothermic migrants accumulate fat over multiple days increasing fat while feeding during the day and losing fat during the inactive period at night (solid Line A). One possibility of a shallow torpor strategy during migration involves a heterothermic species which maintains the same fat accumulation rate during the day but uses shallow torpor at night to reduce energy expended at night and increase net refuelling rate compared to a homeotherm (dashed Line B). Alternatively, at a lower quality stopover site where daytime refuelling rate is lower, the reduction in energy expenditure while using shallow torpor at night results in the same net refuelling rate as the homeothermic migrant at the higher quality stopover site (dotted Line C). Figure adapted from Clerc and McGuire (2021).

Although we focused on the migratory period for this study, endotherms undergo many other energetically stressful periods throughout the annual cycle including breeding (Mainwaring & Hartley, 2013; Vézina & Williams, 2003), moult (Cyr et al., 2008) and overwintering periods (Petit et al., 2013), and the driver of torpor use may vary seasonally in passerines, as it does in hummingbirds (Eberts et al., 2021; Powers et al., 2003). We observed birds that reduced their metabolic rate by up to 41% at high *T*_a_ (≥25°C) during migration indicating shallow torpor could be an important energy‐saving strategy during energetic bottlenecks throughout the year. Species that breed, moult or overwinter at high latitudes and altitudes rarely experience *T*_a_ ≥25°C at night and birds in poor condition may benefit from the energy savings associated with shallow torpor (Calder & Booser, 1973).

Torpor has long been recognized as an important thermoregulatory strategy for small mammals in a variety of circumstances throughout the annual cycle (Geiser, 2004), but aside from hummingbirds (Eberts et al., 2021; Powers et al., 2003; Shankar et al., 2020; Wolf et al., 2020), and a few other specific cases (Brigham, 1992; Doucette et al., 2012; Merola‐Zwartjes & Ligon, 2000), there has been relatively little attention to heterothermy in birds, particularly in passerines (but see Benedetti et al., 2014; Maddocks & Geiser, 1997; McKechnie & Lovegrove, 2002; Wojciechowski & Pinshow, 2009). More than half of extant birds are in the order Passeriformes, with more than 6500 species found in all habitats around the globe (Raikow, 1986; Schmitt & Edwards, 2022). Birds represent an independent evolution of endothermy from mammals, and whereas heterothermy has long been a subject of great interest in ecophysiological investigations of mammals, there has been only limited investigation of birds. Our findings indicate shallow torpor is more widespread than currently recognized among migrating birds, with important energetic, ecological and evolutionary consequences. Our study was limited to small‐bodied, temperate migrant species, but there is a great opportunity to investigate the role of heterothermy more broadly in birds, whether during migration or under other ecological contexts, and comparisons of heterothermic birds and small mammals may be especially revealing.

## AUTHOR CONTRIBUTIONS

Ryan A. C. Leys and Liam P. McGuire conceived the ideas and designed the experiments. Ryan A. C. Leys and Lily Hou collected data. Ryan A. C. Leys analysed the data and wrote the manuscript in consultation with Liam McGuire. All authors approved the final manuscript.

## CONFLICT OF INTEREST STATEMENT

The authors declare no conflict of interest.

## Supporting information


**Table S1.** Age, sex, and total number of animals used in Study 1 and Study 2.
**Table S2.** Results of the minimum body temperature models for each species.
**Table S3.** Minimum metabolic rate ranges and means per species and temperature treatment.
**Table S4.** Results of the metabolic rate models for all species.
**Table S5.** Results of the rate of mass loss models for all species.

## Data Availability

Data available from the Open Science Framework https://osf.io/ema6b/ (Leys et al., 2026).
