# Supplementary material for: Torpor‐assisted migration: Regular use of shallow torpor reduces rest‐phase energy expenditure for songbirds during migration
Source: J Anim Ecol. 2026 Mar 30;95(10):1876–86. doi: 10.1111/1365-2656.70244 (PMC13643193; doi:10.1111/1365-2656.70244)
Supplement: Supplementary file 1 [file JANE-95-1876-s001.pdf]

SUPPLEMENTARY MATERIAL

Table 1. Age, sex, and total number of animals used in study 1 and study 2. Total tested is the number of birds included in the experimental procedures. Some birds were excluded from statistical analysis (see text) resulting in the Total Analyzed number of birds included in the statistical analysis. HY- Hatching year, SY- Second year, AHY- After hatching year, ASY- After second year. Birds with an unknown age and/or sex are omitted from the age/sex breakdown but included in the totals columns.

**Protocol**	**Species**	**Male (HY/SY)**	**Female (HY/SY)**	**Male (AHY/ASY)**	**Female (AHY/ASY)**	**Total Analyzed**	**Total Tested**
**Study 1: Aviary**	Brown Creeper	0	0	0	0	20	20
	Golden-crowned Kinglet	3	9	7	2	21	25
	Myrtle Warbler	10	5	0	2	17	19
	Ruby-crowned Kinglet	21	13	8	7	49	53
**Study 2: Respirometry**	Brown Creeper	0	0	0	0	23	24
	Golden-crowned Kinglet	17	14	3	3	37	41
	Myrtle Warbler	7	18	3	3	35	40
	Ruby-crowned Kinglet	12	14	4	7	37	42

Table 2. Results of the minimum body temperature models for each species. The effect of fat score differed between study 1 and study 2 (study:fat score interaction) for Golden-crowned Kinglet, therefore we analyzed the two studies separately. Model

coefficients are only presented for significant effects ($p < 0.034$) after controlling for the false discovery rate using the Benjamini-Hochberg procedure.

Species	Predictor Variable	Coefficient	p-value	F-value
**Brown Creeper**	T_a		0.05	$F_{1,39} = 4.1$
	Fat score	0.43	**0.001**	$F_{1,39} = 11.7$
	Study	0.87	**0.002**	$F_{1,39} = 10.9$
**Golden-crowned Kinglet**	Age		0.92	$F_{1,51} = 0.01$
	Sex		0.32	$F_{1,53} = 1.0$
	T_a		0.48	$F_{1,52} = 0.5$
	Study * Fat score		**< 0.001**	$F_{1,54} = 14.6$
**Golden-crowned Kinglet (Study 1)**	Age		0.39	$F_{1,16} = 0.8$
	Sex		0.41	$F_{1,17} = 0.7$
	T_a		0.12	$F_{1,18} = 2.6$
	Fat score	1.7	**0.001**	$F_{1,19} = 14.6$
**Golden-crowned Kinglet (Study 2)**	Age		0.78	$F_{1,32} = 0.1$
	Sex		0.65	$F_{1,33} = 0.2$
	T_a		0.51	$F_{1,34} = 0.5$
	Fat score		0.16	$F_{1,35} = 2.1$
**Myrtle Warbler**	Age		0.25	$F_{1,45} = 1.3$
	Sex		0.09	$F_{1,46} = 2.5$
	T_a		0.07	$F_{1,49} = 3.5$
	Fat score	0.44	**< 0.001**	$F_{1,50} = 12.7$
	Study		0.19	$F_{1,48} = 1.8$
**Ruby-crowned Kinglet**	Age		0.32	$F_{1,80} = 1.0$
	Sex		0.036	$F_{1,82} = 4.6$
	T_a		0.045	$F_{1,82} = 4.1$
	Fat score	0.29	**0.001**	$F_{1,82} = 11.6$
	Study		0.28	$F_{1,81} = 1.2$

Table 3. Minimum metabolic rate ranges and means per species and temperature treatment. All mean values reported in mL O_2 min^{-1} ± SE. Golden-crowned Kinglet were not tested at 25°C. Brown Creepers and Myrtle Warblers were not tested at 30°C. We do not report range or mean values for Brown Creeper at 12 °C or Ruby-crowned Kinglet at 25 °C as only two birds were tested at each of these treatment temperatures.

**Species**		**2°C**	**7°C**	**12°C**	**25°C**	**30°C**
**Brown Creeper**	Range	0.85 – 1.13	0.86 – 1.07		0.49 – 0.66	
	Mean	1.07 ± 0.04	0.93 ± 0.03		0.55 ± 0.02	
**Golden-crowned Kinglet**	Range	0.80 – 1.11	0.68 – 0.86	0.50 – 0.87		0.28 – 0.48
	Mean	0.94 ± 0.04	0.81 ± 0.02	0.67 ± 0.04		0.38 ± 0.02
**Myrtle Warbler**	Range	1.09 – 1.47	0.90 – 1.31	0.86 – 1.27	0.60 – 0.87	
	Mean	1.27 ± 0.05	1.11 ± 0.04	1.02 ± 0.04	0.69 ± 0.03	
**Ruby-crowned Kinglet**	Range	0.76 – 1.15	0.90 – 1.11	0.73 – 0.94		0.43 – 0.55
	Mean	0.96 ± 0.04	0.97 ± 0.03	0.81 ± 0.02		0.47 ± 0.01

Table 4. Results of the metabolic rate models for all species. As expected, metabolic rate increased at colder temperatures, but (generally) that effect was mitigated by reduced metabolic rate in birds with lower body temperature and lower fat score. Model coefficients are only presented for significant effects ($p < 0.034$) after controlling for the false discovery rate using the Benjamini-Hochberg procedure.

Species	Predictor Variable	Coefficient	p-value	F-value
**Brown Creeper**	T_a	-0.02	**< 0.0001**	$F_{1,20} = 299.2$
	T_b		0.69	$F_{1,19} = 0.2$
	Fat score	0.05	**0.002**	$F_{1,20} = 13.4$
	Julian Day		0.80	$F_{1,18} = 0.07$
**Golden-crowned Kinglet**	Age		0.89	$F_{1,30} = 0.02$
	Sex		0.74	$F_{1,31} = 0.1$
	T_a	-0.02	**< 0.0001**	$F_{1,33} = 350.1$
	T_b	0.05	**< 0.0001**	$F_{1,33} = 20.1$
	Fat score		0.05	$F_{1,33} = 4.2$
	Julian Day		0.16	$F_{1,32} = 2.0$
**Myrtle Warbler**	Age		0.55	$F_{1,29} = 0.4$
	Sex		0.84	$F_{1,27} = 0.2$
	T_a	-0.03	**< 0.0001**	$F_{1,30} = 573.8$
	T_b	0.05	**< 0.0001**	$F_{1,30} = 42.3$
	Fat score	0.05	**< 0.0001**	$F_{1,30} = 28.4$
	Julian Day	-0.003	**0.001**	$F_{1,30} = 12.4$
**Ruby-crowned Kinglet**	Age		0.91	$F_{1,30} = 0.01$
	Sex		0.91	$F_{1,31} = 0.01$
	T_a	-0.02	**< 0.0001**	$F_{1,34} = 260.7$
	T_b	0.04	**0.03**	$F_{1,34} = 5.3$
	Fat score		0.27	$F_{1,33} = 1.26$
	Julian Day		0.38	$F_{1,32} = 0.80$

Table 5. Results of the rate of mass loss models for all species. The effect of sex differed between study 1 and study 2 (study:sex interaction) for Myrtle Warblers and the effect of percentage of time using torpor differed between study 1 and study 2 for Golden-crowned Kinglets (study:percentage of time using torpor interaction), therefore we analyzed the two studies

separately for each species. Model coefficients are only presented for significant effects ($p < 0.034$) after controlling for the false discovery rate using the Benjamini-Hochberg procedure.

Species	Predictor Variable	Coefficient	p-value	F-value
**Brown Creeper**	T_a		0.34	$F_{1,41} = 0.9$
	Percentage of time using torpor		0.86	$F_{1,39} = 0.03$
	Study		0.51	$F_{1,40} = 0.4$
**Golden-crowned Kinglet**	Age		0.22	$F_{1,53} = 1.5$
	Sex		0.48	$F_{1,52} = 0.5$
	T_a		0.99	$F_{1,51} = 0.0001$
	Percentage of time using torpor * Study		**0.03**	$F_{1,54} = 4.9$
**Golden-crowned Kinglet (Study 1)**	Age		0.51	$F_{1,17} = 0.5$
	Sex		0.79	$F_{1,16} = 0.1$
	T_a		0.35	$F_{1,58} = 0.9$
	Percentage of time using torpor	-0.0002	**0.003**	$F_{1,19} = 11.2$
**Golden-crowned Kinglet (Study 2)**	Age		0.40	$F_{1,35} = 0.7$
	Sex		0.62	$F_{1,33} = 0.3$
	T_a		0.95	$F_{1,32} = 0.004$
	Percentage of time using torpor		0.56	$F_{1,34} = 0.3$
**Myrtle Warbler**	Age		0.32	$F_{1,44} = 1.0$
	T_a		0.15	$F_{1,46} = 2.2$
	Percentage of time using torpor		0.10	$F_{1,45} = 2.9$
	Sex * Study		**0.005**	$F_{1,47} = 8.8$
**Myrtle Warbler (Study 1)**	Age		0.97	$F_{1,12} = 0.002$
	Sex		0.29	$F_{1,15} = 1.2$
	T_a		0.60	$F_{1,14} = 0.3$
	Percentage of time using torpor		0.52	$F_{1,13} = 0.4$
**Myrtle Warbler (Study 2)**	Age		0.38	$F_{1,29} = 0.8$
	Sex		**0.005**	$F_{2,32} = 6.2$
	Female	-0.03	**0.004**	

	Unknown		0.08	
	T_a		0.15	$F_{1,31} = 2.2$
	Percentage of time using torpor		0.10	$F_{1,30} = 2.9$
**Ruby-crowned Kinglet**	Age		0.79	$F_{1,80} = 0.07$
	Sex		0.16	$F_{1,82} = 2.0$
	T_a		0.57	$F_{1,81} = 0.3$
	Percentage of time using torpor	-0.0001	**0.01**	$F_{1,83} = 6.8$
	Study	-0.006	**0.02**	$F_{1,83} = 5.6$
